# Controllable Unsupervised Snow Synthesis by Latent Style Space Manipulation

**DOI:** 10.3390/s23208398

**Published:** 2023-10-12

**Authors:** Hanting Yang, Alexander Carballo, Yuxiao Zhang, Kazuya Takeda

**Affiliations:** 1 Graduate School of Informatics, Nagoya University, Furo-cho, Chikusa-ku, Nagoya 464-8601, Japan; yuxiao.zhang@g.sp.m.is.nagoya-u.ac.jp (Y.Z.); takeda@g.sp.m.is.nagoya-u.ac.jp (K.T.); 2 Faculty of Engineering, Graduate School of Engineering, Gifu University, 1-1 Yanagido, Gifu City 501-1193, Japan; alexander@g.sp.m.is.nagoya-u.ac.jp; 3 Institute of Innovation for Future Society, Nagoya University, Furo-cho, Chikusa-ku, Nagoya 464-8601, Japan; 4 Tier IV Inc., Nagoya University Open Innovation Center, 1-3, Mei-eki 1-chome, Nakamura-Ward, Nagoya 450-6610, Japan

**Keywords:** intelligent vehicles, snow scenes, unpaired image-to-image translation, diversity, style latent space, Gaussian distribution

## Abstract

In the field of intelligent vehicle technology, there is a high dependence on images captured under challenging conditions to develop robust perception algorithms. However, acquiring these images can be both time-consuming and dangerous. To address this issue, unpaired image-to-image translation models offer a solution by synthesizing samples of the desired domain, thus eliminating the reliance on ground truth supervision. However, the current methods predominantly focus on single projections rather than multiple solutions, not to mention controlling the direction of generation, which creates a scope for enhancement. In this study, we propose a generative adversarial network (GAN)–based model, which incorporates both a style encoder and a content encoder, specifically designed to extract relevant information from an image. Further, we employ a decoder to reconstruct an image using these encoded features, while ensuring that the generated output remains within a permissible range by applying a self-regression module to constrain the style latent space. By modifying the hyperparameters, we can generate controllable outputs with specific style codes. We evaluate the performance of our model by generating snow scenes on the Cityscapes and the EuroCity Persons datasets. The results reveal the effectiveness of our proposed methodology, thereby reinforcing the benefits of our approach in the ongoing evolution of intelligent vehicle technology.

## 1. Introduction

Intelligent vehicles and other advanced mobile agents are engineered to navigate through a spectrum of adverse weather conditions. This poses a formidable challenge to perception algorithms [1]. To enhance the robustness of these algorithms, a prevalent strategy involves augmenting the training dataset [2,3,4]. However, operating vehicles under such severe conditions contravenes road safety regulations, and data acquisition, in this case, becomes significantly time-consuming.

A viable alternative to traditional data collection is to synthesize weather effects on existing public benchmarks [5,6,7,8,9]. Conventionally, this is accomplished by modeling the impact of weather effects, such as fog, rain, and snow, as a function [10]. The derived function is subsequently applied to images or videos, thus simulating the desired weather conditions. This method facilitates the creation of diverse datasets, which serve as valuable resources for training and testing various perception algorithms, encompassing object detection, intention estimation, trajectory prediction, etc.

In light of the widespread adoption of deep learning methodologies, several researchers have begun considering the use of physical synthesis datasets as sources to train more universally applicable convolutional neural networks (CNNs) or generative adversarial networks (GANs) [11,12]. These datasets furnish diverse and realistic training samples, without the need for actual data collection in perilous weather conditions. Still, the efficacy of this approach highly depends on the accuracy of the weather effect model and the quality of the synthesized images or videos.

A recently proposed concept involves utilizing unpaired image-to-image translation models. This type of model is capable of learning how to map visual features from a source domain to a target domain without one-to-one correspondence [13,14,15]. A prominent example in this domain is the CycleGAN architecture [14], designed to generate images based on GANs that are virtually indistinguishable from real photographs. The key innovation introduced through CycleGAN is the implementation of a cycle consistency loss. This feature encourages the mapping of an image from one domain to another to be consistent and vice versa. Consequently, the model is able to learn a mapping between two image collections, effectively capturing correspondences between higher-level appearance structures.

In our previous research [16], we implemented a CycleGAN-based model to synthesize realistic snow on driving scene images. We used the Cityscapes and EuroCity Persons datasets as source domains, while a self-captured snow collection functioned as the target. The image generation performance was assessed using a variety of image quality metrics. Benefiting from semantic information, each sample was effectively transformed into convincing snow scenes, while maintaining the integrity of the original image’s structure and texture. However, the adopted method yielded a single output conditioned on the given input image, which does not fully leverage the inherent multimodality of the mapping between two visual domains. This limitation overlooks the potential diversity of snow scenes that may be present.

**Figure 1:**
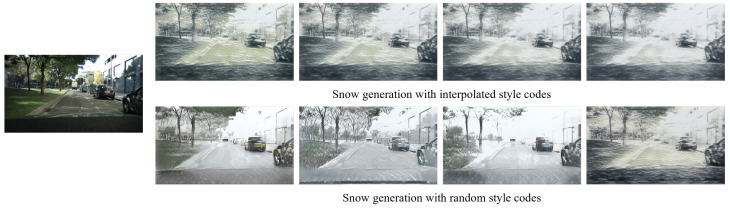
Examples of the output produced by the proposed snow synthesis model. In the upper row, the dimensions of the snow are progressively enlarged, an effect accomplished through the interpolation of style codes. Conversely, the images in the lower row are generated using randomly sampled style codes that follow a Gaussian distribution.

In the present study, we present a novel framework, controllable unsupervised snow synthesis (CUSS), devised to overcome the limitations inherent in existing snow synthesis methodologies. The reason we focus on snow is that snow can drastically reduce visibility, often more than rain or haze, and accumulating snow will cover the road surface and points of interest. The novelty of this work stems from the presumption that the snow representation can be decomposed into a texture-invariant content code and a snow-specific style code. Further, in the middle of the training process, we explore the latent space by a self-regression module. The module linearly interpolates the style code of the clear domain and the snow domain. After training, we can twist the style code with a hyperparameter that theoretically controls the size of the snow as shown in the Figure 1. This strategy facilitates a more comprehensive capture of the full distribution of potential outputs, marking a significant advancement within the domain. The key contributions of our research are as follows:

- Content and style disentanglement. The CUSS model employs an architectural framework comprising a content encoder and a style encoder. In order to separate the content and style latent spaces, we introduce a supplementary content discriminator that distinguishes the content codes of clear and snow images.
- Multimodal controllable output generation. The CUSS model allows for the generation of multiple and diverse outputs based on a single input image through the sampling of distinct style codes. Moreover, the incorporation of a self-regression module facilitates the linear interpolation of the style code, thereby enabling manual adjustment of the generated size of snow.
- Evaluations on public datasets. The model undergoes evaluation employing the Cityscapes and EuroCity Persons datasets. Various image quality metrics, encompassing the traditional PSNR, SSIM, and perceptual loss VGG distance, are employed to substantiate the effectiveness of the model. The source code will be available on https://github.com/HantingYang/Controllable-Snow-Synthesize (accessed on 13 September 2023).

## 2. Related Work

### 2.1. Weather Generation

Despite extensive research on improving visibility during inclement weather, there has been limited attention given to incorporating artificial weather effects into existing driving datasets. Our literature review delves into the current techniques for synthesizing weather, particularly those leveraging deep learning. Utilizing a generative model for weather simulation could offer a versatile instrument for generating authentic conditions to evaluate and enhance AI-driven systems.

#### 2.1.1. Fog and Rain

The fog generation process, as detailed in a study conducted by Christos et al. [6], comprises two primary steps. It is initialized with the estimation of a transmission map and atmospheric light based on a clear scene image. Following this, depth denoising and completion techniques are implemented to enhance the accuracy of the depth map. This refined depth map is subsequently employed to simulate fog, culminating in the generation of synthetic fog images that closely resemble real-world foggy scenarios. Zhang et al. [17] introduce a technique to generate haze images from clear ones, employing a network that includes two encoders with shared feature extraction layers. The essence of their approach lies in separating the style feature, used exclusively for haze synthesis, from the content feature that conveys consistent semantic information. The authors control a parameter α to synthesize multidensity haze images, with the networks learning to distinguish between thick and thin haze.

For the rain generation, Garg and Nayar [7] propose an adaptive image-based rendering technique that utilizes a database of rain streak appearances, requiring only the positions and attributes of light sources and a rough depth map, to add photorealistic rain to a single image or a recorded video with moving objects and sources. Venceslas et al. [18] discuss a real-time rendering algorithm for the realistic representation of fog in animations. The algorithm works by rendering fog for each pixel of the screen. The authors also introduce the concept of equipotential functions, which allow for the creation of complex shapes of fog.

#### 2.1.2. Snow

Liu et al. [8] suggest a snow synthesis method that utilizes base masks representing different snow particle sizes—small, medium, and large. The snow synthesis process involves overlaying these base masks with images and introducing random factors such as snow brightness and random cropping to increase variation.

Ohlsson et al. [19] present a real-time method for rendering accumulated snow, which involves determining which regions should receive snow based on surface inclination and exposure to the sky, and then rendering the snow convincingly at those locations. The rendering process uses a Phong illumination model and a noise function to distort the surface normally, creating a realistic snow cover appearance and an illusion of snow depth around occlusion boundaries. The exposure function is implemented using a depth buffer, similar to shadow mapping, and the depth map is sampled multiple times to calculate a fractional value for occlusion, creating a smooth transition between snow-covered and non-snow-covered areas.

Alexey et al. [20] introduce an innovative approach for snow simulation through a user-adjustable elastoplastic constitutive model paired with a hybrid material point method (MPM). The MPM employs a consistent Cartesian grid to facilitate self-collision and fracture automatically. Moreover, it utilizes a grid-centric semi-implicit integration scheme not reliant on the count of Lagrangian particles. This technique adeptly simulates diverse snow behaviors, especially the intricate dynamics of dense and wet snow, and incorporates rendering methods for a true-to-life visual depiction of snow.

As an evaluation work, Thomas et al. [5] take multiple cutting-edge image-to -mage (I2I) translation models for comparison and CycleGAN [14] as the baseline. The models used include UNIT [21] and MUNIT [22]. This work attempts to generate all kinds of bad weather images; the main focus is snow scenes. Authors believe that the identity loss that is calculated by the Manhattan distance between input and reconstructed images plays an essential role in the translation process. Therefore, they train the model several times and each time specify a different weight for the loss. In addition, to make up for the shortness of current datasets, images retrieved from the image engine Flickr are fed to their model.

Our previous work [16] presents a novel method for synthesizing realistic snow images on driving datasets using cycle-consistent adversarial networks. We introduce a multimodality module that uses a segmentation map to accurately generate snow according to different regions of an image. We also propose a deep supervision module that adds extra side outputs to the discriminator, improving the network’s learning of discriminative features. The model is evaluated using the same loss functions as CycleGAN [14]. The evaluation results on the Cityscapes and EuroCity Persons datasets show that the model outperforms other methods in generating realistic snow images.

From the above, generative models such as GANs are able to generate scenes under a variety of challenging conditions and make the output convincible. Models based on cycle consistency are able to generate images from the target domain without paired data. However, these models can only produce one output based on one input, and the degree of style transfer cannot be controlled. This work aims to further explore the latent space of extracted features and make the model produce diverse results.

### 2.2. Unpaired Image-to-Image Translation

Image-to-image (I2I) translation focuses on learning the mapping between two domains [23,24]. This involves capturing correspondences between higher-level appearance structures. The goal is to transform an image from a source domain to a target domain while preserving the underlying structure or context. Unpaired I2I translation further improves the training process, and it does not require paired input–output examples [13,14,15]. Instead, it assumes that there is some underlying relationship between the two domains and seeks to learn that relationship. This approach is particularly useful when image pairs are unavailable or the sensing environment is dangerous like driving scenes under challenging conditions.

#### Latent Space Constraint

With the success of unpaired I2I translation, researchers are now directing their attention to generating a more diverse range of output. This is achieved by a latent space constraint.

Zhu et al. [25] introduce the BicycleGAN model, designed to enhance image-to-image translation by producing diverse and realistic outcomes using a concise latent vector, and this model utilizes a combined technique that ensures a one-to-one consistency between latent encoding and output modes to avoid mode collapse. In their research, they explored various methods of incorporating the latent code into the generator and observed similar performance levels while also investigating the balance between result diversity and sampling complexity by adjusting the latent code’s dimensionality.

Lee et al. [26] suggest a technique that projects input images into a joint content space and domain-distinct attribute areas. The content encoders relay the mutual details shared across domains to shared content space, and attribute encoders relay the domain-unique data to specific attribute space. To handle datasets without pairs, they introduced a cross-cycle consistency loss leveraging the separate representations.

Huang et al. [22] showcase the multimodal unsupervised image-to-image translation (MUNIT) structure, which differentiates image representation into a universal content code and a domain-centered style code, and incorporates two autoencoders, competitive objectives, and two-way reconstruction objectives to produce a range of results from a single source image. Furthermore, the model introduces the concept of style-enhanced cycle consistency, ensuring that the original image is recovered when converted to a target domain and reverted using its initial style.

Choi et al. [27] discuss a unified model called StarGAN that handles I2I translations across multiple domains. The generator takes an input image and a target domain label to generate a fake image. This target domain label is represented as a binary or one-hot vector for categorical attributes. The generator focuses on the explicitly given label and ignores unspecified labels by setting zero vectors, enabling the model to generate high-quality images.

Liu et al. [28] introduce a technique named the unified feature disentanglement network (UFDN) designed for self-supervised feature decomposition. They utilize a variational autoencoder (VAE) structure to achieve disentangled representations across various data domains. The encoder accepts an image, processes its representation, and then merges it with the domain vector. These combined data are then used by the generator to recreate the image.

Inspired by the above work, we introduce a content and style representation from our previous snow synthesis framework [16]. For intuition, we divide the translation step into three parts: encoding, translation, and decoding. The encoding network encodes the input image into one style code and one content code. By swapping and twisting the style code generated by the style encoder, we can obtain diverse but high-quality outputs. In addition, we interpolate the style code between the clear and snow domains to obtain gradually increasing snow effects. This is achieved by disentangling the latent space.

## 3. Controllable Unsupervised Snow Synthesis

To synthesize realistic snow on the driving datasets, we focus on the GAN with cycle consistency. The goal is to learn the mapping between the snow domain and the clear weather domain. In our previous unpaired I2I methods [29,30], two generators are employed to transfer images into the expected domain. Two corresponding discriminators are employed to differentiate real images and fake images. The cycle consistency ensures that translated images can be reconstructed into original input images.

Recently, when researchers use similar methods for weather removal or synthesis, they follow an assumption that weather images can be decomposed into a content partition and a weather partition [17,31,32]. The partition could be any mathematical format, such as vectors or tensors. In general image translation tasks, the weather partition refers to the style representation. This technique will disentangle the translation process and preserve the structural feature of the background. Therefore, we follow the assumption and split the generator into three networks, which are a style encoder, a content encoder, and a decoder.

In the field of representation learning, incomplete disentanglement is often more prevalent. This concept suggests that images from varying domains have a shared content representation space, but the style representation space remains unique to each domain. This idea is also known as the shared latent space assumption. In our task, the style is related to snow, and different classifications detail the attributes of weather events that produce snow.

Intuitively, the content codes and style codes should be disjoint in the representation space. To better achieve representation disentanglement, we apply a content discriminator to distinguish the domain membership of the encoded content features. The goal is to force content encoders to generate features that cannot be identified, which means the content code does not contain style details.

Further, in order to make the size of synthesized snow controllable, we need to explore the space of style partition *S*. Inspired by the work of Zhang et al. [17], we transform the snow domain into a continuous space by associating the style code vectors with a linear manipulation. With the help of the content discriminator, the style code will not contain information on image attributes. Ideally, the interpolated style code should represent an intermediate snow density.

### 3.1. Fundamental Basis

To illustrate the framework of controllable unsupervised snow synthesis (CUSS) in an intuitive way, suppose that $x_{1}\in X_{1}$ and $x_{2}\in X_{2}$ are images from the clear domain and the snow domain, respectively. In statistics, the images belong to two marginal distributions, $p(x_{1})$ and $p(x_{2})$. The joint distribution $p(x_{1},x_{2})$ is inaccessible due to a lack of paired data. The goal is to learn an I2I translation model that can estimate two conditionals, $p(x_{1\to 2}|x_{1})$ and $p(x_{2\to 1}|x_{2})$, where $x_{1\to 2}$ is a sample of synthesized snow images and $x_{2\to 1}$ is a sample of synthesized clear images (recovered from real snow samples). In general, the synthesis outputs do not fall into a single mode. There are multiple solutions corresponding to the transform problem.

To obtain other possible solutions, we adopt the partially shared latent space assumption from MUNIT [22] to produce diverse snow effects. This theory posits that each image $x_{i}\in X_{i}$ originates from a content latent code $c_{i}$, shared across both domains and a unique style latent code $s_{i}$ tied to its respective domain. For snow synthesis, a matching pair of clear and snow images $\left(x_{1},x_{2}\right)$ from the combined distribution is created by $x_{1}=F_{1}\left(c_{1},s_{1}\right)$ and $x_{2}=F_{2}\left(c_{2},s_{2}\right)$, with $F_{1},F_{2}$ as the foundational generators with the inverse encoders $E_{1}$ and $E_{2}$, with $E_{1}={\left(F_{1}\right)}^{-1}$ and $E_{2}={\left(F_{2}\right)}^{-1}$.

**Figure 2:**
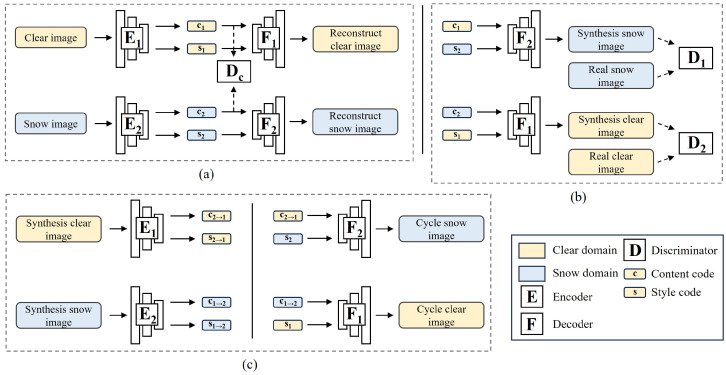
The architectural design of the proposed controllable unsupervised snow synthesis (CUSS) network is outlined as follows. The solid arrows show the forward process of the generators. The dashed arrows show the input to the discriminators. CUSS comprises two encoders, which assume the responsibility of encoding images from both the clear and snow domains, yielding a content code and a style code, respectively. Additionally, CUSS incorporates two decoders, which accept a content code and a style code as input, subsequently generating synthetic images pertaining to the target domain. Moreover, there exist two discriminators, whose purpose is to discern images originating from each domain, alongside a content discriminator, which endeavors to discriminate between content codes. (**a**) illustrates the fundamental pipeline, wherein the encoded images ought to be recoverable utilizing identical codes. Conversely, (**b**) portrays the process of translation accomplished by substituting the style code with a randomly sampled one. Lastly, (**c**) exemplifies the translation process’s cycle consistency, whereby the translated synthetic images ought to revert to the original input, utilizing the initially extracted style code in conjunction with their own content code.

The structure of the CUSS model is depicted in Figure 2. As displayed in Figure 2a, our conversion model has an encoder $E_{1}$ and a decoder $F_{1}$ for the clear domain $X_{1}$, and an encoder $E_{2}$ and a decoder $F_{2}$ for the snow domain $X_{2}$. Each image fed into the encoder becomes converted into a content code *c* and a style code *s*, represented as (c,s)=E(x). The translation between images occurs by interchanging encoder–decoder pairs, as depicted in Figure 2b. For instance, to transform a clear image $x_{1}\in X_{1}$ to $X_{2}$, we first capture its content latent code $c_{1}=E_{1}^{c}\left(x_{1}\right)$ and draw a style latent code $s_{2}$ from the normal distribution $q\left(s_{2}\right)∼N\left(0,I\right)$. Then, we employ $F_{2}$ to generate the ultimate snow image $x_{1\to 2}=F_{2}\left(c_{1},s_{2}\right)$.

In earlier research [16], we harnessed the cycle consistency loss [14], measured by the L1 norm of the input image. This aimed to deter the secondary generator from producing arbitrary target domain images. However, Huang et al. [22] demonstrated that if cycle consistency is imposed, the translation model becomes deterministic. As a result, we integrated a style-enhanced cycle consistency in the image-style joint spaces, which aligns better with multimodal image conversion. As illustrated in Figure 2c, we derive the content code $c_{1\to 2}$ and style code $s_{1\to 2}$ from the synthetic snow image $x_{1\to 2}$. We then feed the content code $c_{1\to 2}$ and the identical style latent code $s_{2}$ to the clear decoder $F_{1}$. The result image is named cycle clear image $x_{1\to 2\to 1}$. The idea behind style-enhanced cycle consistency is that by translating an image to a target domain and then back with the original style, we should retrieve the initial image. We do not apply explicit loss measures to ensure this style-enhanced cycle consistency, but it is suggested by the bidirectional reconstruction loss. We show the pseudo-code of CUSS in Algorithm 1.
**Algorithm 1:** Controllable Unsupervised Snow Synthesis (CUSS)   **Input**: Training data pairs $\left(X_{1},X_{2}\right)$           ▹ In order of clear and snow   **Output**: Encoders $E_{1},E_{2}$, Decoders $F_{1},F_{2}$           ▹$F_{1}$ generate clear images, $F_{2}$ generate snow images  1:Initialize encoders, decoders, and discriminators  2:Define loss functions  3:Define optimizers for generator and discriminator  4:**while** epoch≤total_epoches **do**  5:      **for** data pair ($X_{1}$, $X_{2}$) **in** data_loader **do**  6:          Get content codes and style codes of input images: $\left(c_{1},s_{1}\right)=E_{1}\left(x_{1}\right)$, $\left(c_{2},s_{2}\right)=E_{2}\left(x_{2}\right)$, $\left(s_{n1},s_{n2}\right)∼N\left(0,1\right)$ ▹$s_{n_{i}}$ means style code sampled from normal distribution  7:            Generate fake images: $x_{1\to 2}=F_{2}\left(c_{1},s_{n1}\right)$, $x_{2\to 1}=F_{1}\left(c_{2},s_{n2}\right)$  8:            Generate reconstruct images: $x_{1\to 1}=F_{1}\left(c_{1},s_{1}\right)$, $x_{2\to 2}=F_{1}\left(c_{2},s_{2}\right)$  9:            Get content codes and style codes of fake images: $\left(c_{21},s_{21}\right)=E_{1}\left(x_{2\to 1}\right)$, $\left(c_{12},s_{12}\right)=E_{2}\left(x_{1\to 2}\right)$10:            Generate cycle translation images: $x_{1\to 2\to 1}=F_{1}\left(c_{12},s_{1}\right)$, $x_{2\to 1\to 2}=F_{2}\left(c_{21},s_{2}\right)$11:            **Update** Discriminator $D_{1}$, $D_{2}$, and $D_{c}$12:            **Update** Generator $E_{1}$, $E_{2}$, $F_{1}$, and $F_{2}$13:      **end for**14:**end while**

### 3.2. Disentanglement of Content and Style

A disentangled representation captures the underlying structure of the data so that individual factors can be modified independently without affecting others. The goal is to achieve complete disentanglement, where both content and style features are extracted independently. To achieve this, a content discriminator $D_{c}$ is used to remove style information from the content feature. At the same time, we use self-supervised style coding to reduce content information from the style feature.

To enhance the content encoder, we employ the content feature discriminator proposed by Lee et al. [26]. Initially, the content encoder extracts content codes, denoted as $c_{1}$ and $c_{2}$, from the respective inputs $x_{1}$ and $x_{2}$. The content discriminator $D_{c}$ takes input images and classifies their source domain. Then one objective of the content encoder is to deceive $D_{c}$ with distinct features. As a result, the content encoder and discriminator refine each other through adversarial training. Once equilibrium is reached, the content features extracted no longer retain any stylistic information of the image.

When this game of generators and discriminators stabilizes at the Nash equilibrium [33], it becomes impossible for $D_{c}$ to ascertain the image domain of the content feature, implying an absence of snow details in the content feature. A successful separation of style from content is achieved when the content encoder exclusively captures the image’s content characteristics.

**Figure 3:**
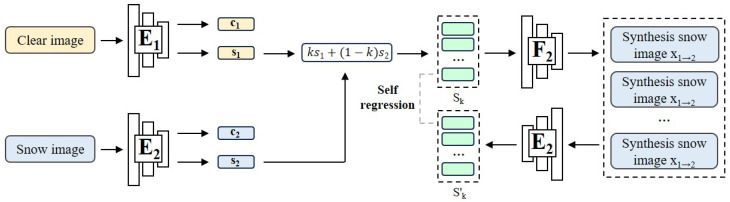
Snow sizing through self-regression style coding. Our methodology involves extracting style codes from two distinct domains. By using linear interpolation between these domains, guided by the parameter *k*, we are able to generate a range of snow sizes. A higher value of *k* gives greater significance to the snow dimensions. We then merge the content and interpolated style features to create a snow scene image with a specific snow density determined by the aforementioned parameter *k*. Throughout the training process, we use a randomly selected *k* value from the interval [0, 1] to derive a novel style code that represents an intermediate level of snow density. This newly generated style code serves as a self-supervised pseudo-label, effectively guiding the updating process of the style encoder.

It is proved that utilizing the content discriminator can prevent content codes from containing style details [26]. Naturally, the next step is to remove content details from style codes. The purpose is to make the generation more stable without being affected by other factors. Consequently, we implement the self-supervised style coding to remove any excess content details from the style codes, illustrated in Figure 3.

Using a nonlinear function *f* to denote the style encoder, we initially perform interpolation on two style codes, one from the clear domain and the other from the snow domain to acquire $s_{k}$, as shown in Equation (1).

(1) $$s_{k}=f\left(kx_{1}+\left(1-k\right)x_{2}\right)$$

In the scope of our problem, the need to disentangle the style feature from the content feature makes sure that the operation on the style code is consistent with those on the input image.

According to the derivation of Zhang’s work [17], because $s_{k}$ is calculated by the linear projection of $s_{1}$ and $s_{2}$, it should contain snow detail that is also a linear relation of $x_{1}$ and $x_{2}$. Use $s_{k}$ and a content code of a clear input $c_{1}$ and a new snow image $x_{k}$ can be generated. We then encode $s_{k}$ again to obtain its style code, which will be supervised by $s_{k}$ itself; the loss function is defined as Equation (2).

(2) $$\begin{array}{cc} \; & L_{s}\left(E_{1}^{c},E_{2}^{s},F_{2}\right)=\\ \; & E_{x_{1},x_{2}}\left[{∥E_{2}^{s}\left(F_{2}\left(E_{1}^{c}\left(x_{1}\right),s_{k}\right)\right)-s_{k}∥}_{1}\right. \end{array}$$

Even though the function *f* is nonlinear, we continually generate $x_{k}$ and optimize the encoder with $s_{k}$ in the training phase to maintain a consistent relationship between input images and corresponding style codes linearly.

In the early stages, the style code will contain an extra content detail because of latent space entanglement. At every forward iteration, the extra details become separated with the style codes. Instinctively, the style encoder will identify content details and ignore them.

In the absence of manually assigned labels, the process relies on $s_{k}$ as a self-generated label to guide the updates of networks. The desired situation is that the style code will generate snow according to each object distribution at every distance and not decrease the information density of traffic sign areas.

Due to the stochastic choice of *k* at each forward iteration, the encoder is compelled to project the snow-related detail into a linear space. As a result, we engage in linear adjustments to style codes to generate images with varying snow densities.

For example, we can specify the *k* value presenting the k×100% snow density of the input snow image. Then we extract the style code and content code of the input clear image. After that, we feed the content code and interpolate the style code to the decoder to obtain the output.

The factor *k* governs the snow density. Since $s_{2}$ originates from the baseline snow, it can be scaled up or down using *k* to yield a background invariant image featuring different levels of snow density as depicted in Equation (3).

(3) $$x_{k}=F_{2}\left(E_{1}^{c}\left(x_{1}\right),kE_{1}^{s}\left(x_{1}\right)+\left(1-k\right)E_{2}^{s}\left(x_{2}\right)\right)$$

### 3.3. Loss Function

The comprehensive loss function discussed in this paper comprises several components: the adversarial loss $L_{adv}$, image reconstruction identity loss $L_{id}$, style reconstruction loss $L_{recon}^{s}$, content reconstruction loss $L_{recon}^{c}$, style regression loss $L_{regre}$, cycle consistency loss $L_{cc}$, and content loss $L_{cont}$. The overall objective function is formulated as the weighted sum of these individual loss components:

(4) $$\begin{array}{cc} L= & \lambda_{adv}L_{adv}+\lambda_{id}L_{id}+\lambda_{\mathrm{recon}}^{s}L_{\mathrm{recon}}^{s}+\lambda_{\mathrm{recon}}^{c}L_{\mathrm{recon}}^{c}\\ & +\lambda_{\mathrm{regre}}^{s}L_{\mathrm{regre}}^{s}+\lambda_{cc}L_{cc}+\lambda_{cont}L_{cont} \end{array}$$

Here, $L_{adv}=L_{D_{1}}+L_{D_{2}}$. $L_{D}$ represents the adversarial losses in the clear and snow images. The various λ terms act as the model’s hyperparameters, modulating the significance of each loss component.

#### 3.3.1. Adversarial Loss

Adversarial loss is employed in both the clear and snow domains to enhance the realism of the generated images. In the domain of clear images, the adversarial loss is specified as follows:

(5) $$\begin{array}{cc} L_{D_{1}} & =E_{x_{1}∼P_{X_{1}}}\left[\log D_{1}\left(x_{1}\right)\right]\\ & +E_{x_{2}∼P_{X_{2}}}\left[\log\left(1-D_{1}\left(F_{1}\left(E^{c}\left(x_{2}\right),s_{1}\right)\right)\right)\right] \end{array}$$

$D_{1}$ serves the purpose of differentiating real clear images from their synthesized counterparts, striving to maximize the aforementioned loss function. On the other hand, $F_{2}$ aims to reduce the loss in order to make the generated clear images appear more authentic. Likewise, $L_{D_{2}}$ for the snow domain is defined as

(6) $$\begin{array}{cc} L_{D_{2}} & =E_{x_{2}∼P_{X_{2}}}\left[\log D_{2}\left(x_{2}\right)\right]\\ & +E_{x_{1}∼P_{X_{1}}}\left[\log\left(1-D_{2}\left(F_{2}\left(E^{c}\left(x_{1}\right),s_{2}\right)\right)\right)\right] \end{array}$$

We consider both adversarial losses to have equal impact and straightforwardly sum them up to compose the ultimate adversarial loss.

(7) $$L_{adv}=L_{D_{1}}+L_{D_{2}}$$

#### 3.3.2. Identity Loss and Latent Space Reconstruction Loss

When provided with a snow image and a clear image, the encoders are required to recreate the input image based on the same content code and style code. As such, the disparity between the reassembled image and the initial image serves as the reconstruction loss, adding additional constraints to the encoder:

(8) $$\begin{array}{cc} L_{\mathrm{id}} & =E_{x_{1}∼P_{X_{1}}}\left[{∥F_{1}\left(E_{1}^{c}\left(x_{1}\right),E_{1}^{s}\left(x_{1}\right)\right)-x_{1}∥}_{1}\right]\\ \; & +E_{x_{2}∼P_{X_{2}}}\left[{∥F_{2}\left(E_{2}^{c}\left(x_{2}\right),E_{2}^{s}\left(x_{2}\right)\right)-x_{2}∥}_{1}\right] \end{array}$$

Additionally, we aim for the decoded images to have content and style features that closely resemble those in the original images. As a result, we define the following losses for the reconstruction of content code and style code:

(9) $$\begin{array}{cc} L_{\mathrm{recon}\;}^{c} & =E_{x_{1}∼P_{X_{1}}}\left[∥E_{1}^{c}\left(x_{2\to 1}\right)-E_{1}^{c}\left(x_{1}\right){∥}_{1}\right]\\ \; & +E_{x_{2}∼P_{X_{2}}}\left[∥E_{2}^{c}\left(x_{1\to 2}\right)-E_{2}^{c}\left(x_{2}\right){∥}_{1}\right] \end{array}$$

(10) $$\begin{array}{cc} L_{\mathrm{recon}\;}^{s} & =E_{x_{1}∼P_{X_{1}}}\left[∥E_{1}^{s}\left(x_{2\to 1}\right)-E_{1}^{s}\left(x_{1}\right){∥}_{1}\right]\\ \; & +E_{x_{2}∼P_{X_{2}}}\left[∥E_{2}^{s}\left(x_{1\to 2}\right)-E_{2}^{s}\left(x_{2}\right){∥}_{1}\right] \end{array}$$

It is important to note that we treat the reconstruction loss of a style code as falling under the umbrella as the self-supervised style coding loss. We sum these two up, applying the same weight to both, to arrive at the final style coding loss:

(11) $$L_{\mathrm{recon}}^{s}=L_{\mathrm{recon}\;}^{s}+L_{s}$$

### 3.4. Cross-Cycle Consistency Loss

Our model incorporates the cross-cycle consistency loss, as referenced in [26], to facilitate the learning of domain mappings. For the generated snow image $x_{1\to 2}$, its corresponding clear image $x_{2}$ can be recovered through a desnowing transformation. The cross-cycle consistency loss constrains the scope of the generated image while maintaining the background information of the input images. The Manhattan distance between the cyclically reconstructed image and the original image serves as the measure for this cross-cycle consistency loss. The image conversion process, which involves converting the clear image to the snow image and the other way around, proceeds in Equation (12):

(12) $$\begin{array}{cc} \; & x_{1\to 2}=F_{2}\left(E_{1}^{c}\left(x_{1}\right),E_{2}^{s}\left(x_{2}\right)\right)\\ \; & x_{2\to 1}=F_{1}\left(E_{2}^{c}\left(x_{2}\right),E_{1}^{s}\left(x_{1}\right)\right) \end{array}$$

The reverse translation operation, which entails reconstructing the original input from the generated image, is outlined in Equation (13):

(13) $$\begin{array}{cc} \; & x_{1\to 2\to 1}=F_{1}\left(E_{2}^{c}\left(x_{1\to 2}\right),E_{1}^{s}\left(x_{2\to 1}\right)\right)\\ \; & x_{2\to 1\to 2}=F_{2}\left(E_{1}^{c}\left(x_{2\to 1}\right),E_{2}^{s}\left(x_{1\to 2}\right)\right) \end{array}$$

The formulation of the cross-cycle consistency loss for both the snow and clear image domains is

(14) $$\begin{array}{cc} L_{cc} & =E_{x_{1}∼P_{X_{1}}}\left[{∥x_{1}-x_{1\to 2\to 1}∥}_{1}\right]\\ \; & +E_{x_{2}∼P_{X_{2}}}\left[{∥x_{2}-x_{2\to 1\to 2}∥}_{1}\right] \end{array}$$

## 4. Experiments

To validate the efficacy of the approach described in this paper, this section delves into the influence of various modules and loss functions on the generated outcomes. It also benchmarks these outcomes against existing methods through both quantitative and qualitative metrics. Initially, we provide an overview of the datasets used and the implementation specifics of the approach. Subsequently, we offer an in-depth examination of our model, comparing it with current methodologies in the field. We further support the model’s effectiveness by showcasing visualizations of intermediate outcomes and conducting a generalization analysis. The final portion of this section focuses on ablation studies to scrutinize the model’s components. All testing and experimentation are performed on an NVIDIA RTX A6000 GPU equipped with 24 GB of memory.

### 4.1. Datasets

#### 4.1.1. Urban Sceneries: Cityscapes

The Cityscapes collection [34] consists of 5000 detailed images showcasing urban landscapes, predominantly captured in various German cities during daylight hours. This dataset, which primarily focuses on street vistas, intersections, and vehicular scenes, has gained significant popularity for training and evaluating machine vision systems. We use all images as a clear source to train the model, as the number of images is close to our snow set.

#### 4.1.2. European Urban Scenes: EuroCity Persons

The EuroCity Persons collection [35] comprises a vast array of photographs depicting pedestrians, cyclists, and other moving figures within city traffic scenarios. These images were captured from a mobile vehicle across 31 cities in 12 European nations. Each image in this collection is accompanied by a comprehensive set of precise annotations, including bounding boxes around pedestrians and cyclists, as well as additional information, such as direction, visibility, and potential obstructions. This extensive collection is further divided into separate segments for daylight and nighttime scenes, encompassing a grand total of over 47,300 images. To maintain consistency with the snow dataset, we handpicked 5921 snapshots from the daytime training segment.

#### 4.1.3. Snow Condition Driving Dataset

**Figure 4:**
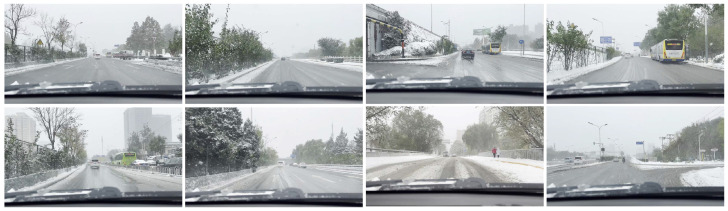
A collection of self-captured videos depicting urban driving amidst intense snowfall [30]. The images are carefully screened. The footage includes various road users, including cyclists, automobiles, buses, and pedestrians.

In order to provide an authentic and realistic benchmark for learning, we recorded a comprehensive video during adverse snowfall conditions. Employing a high-resolution camera positioned behind the windshield of the vehicle, we captured footage at an impressive frame rate of 120 frames per second. From this extensive collection, we carefully selected the highest-quality images and resized them to a resolution of 960×540. Some of the examples are shown in the Figure 4. As a result, our curated snow dataset [30] comprises a total of 6814 meticulously curated photographs. The number of intercepted images we keep is the same as for cityscapes because GAN training is prone to problems such as mode collapse, which leads to training failure.

### 4.2. Implementation Details

Our proposed model’s network comprises two encoders, two decoders, two discriminators, and a content encoder. Among the encoders, one is designed for style, and the other for content. Their structure aligns with what is described in [26]. Breaking it down,

- The content encoder has five convolutional layers.
- The style encoder includes an initial residual layer, two downsampling layers, and one adaptive average pooling layer.
- The content encoder features an initial residual layer, two downsampling layers, and four residual blocks.
- Each decoder is made up of four residual blocks and two upsampling layers. It employs adaptive instance normalization, while the encoders use standard instance normalization.
- All the discriminators take specific image patches with the same resolution as input, which is inspired by Demir’s work [36]. This structure includes five convolutional layers.

For training, we implement minibatch stochastic gradient descent with a batch size of 12, and the Adam optimization technique (parameters: $\beta_{1}=0.5,\beta_{2}=0.999$). We initiate with a learning rate of 0.0001, reducing it linearly from the 100th epoch. In the training phase, the input is cropped to a 256×256 resolution for input. The weight of each loss function is listed below: $\lambda_{adv}=1,\lambda_{id}=10,\lambda_{recon}^{c}=1,\lambda_{recon}^{s}=1\lambda_{regre}^{s}=1,\lambda_{cc}=1$.

### 4.3. Performance Assessment

In this section, we present a comprehensive analysis comparing the outputs CUSS with the current state-of-the-art (SOTA) I2I transition methods. We delve deeply into the impact of disentanglement and conclude with a meticulous examination of the uniqueness and significance of each module based on ablation studies.

#### 4.3.1. Assessment Criteria

Initially, we evaluate the quality of image synthesis using traditional computer vision measures, namely, the peak signal-to-noise ratio (PSNR) and the structural similarity index (SSIM). Additionally, we employ metrics that specifically focus on the depth and the perception features of the images, including the Fréchet inception distance (FID) [33], the LPIPS distance [37], and the VGG distance [38].

PSNR serves as a reliable objective measure for images, quantifying the discrepancy between corresponding pixel values. Higher PSNR values indicate reduced distortion in the generated images.

SSIM, on the other hand, assesses the similarity between two images by considering their luminance, contrast, and structure. An SSIM value of 1 indicates that the two compared images are identical in terms of structural information and quality, while a value of 0 indicates that the images are entirely different in these respects.

FID calculates the Fréchet distance between two sets of images based on the features extracted by the inception network [39]. It provides a measure of similarity between the generated images and their respective benchmarks. A lower FID value suggests that the generated images closely resemble the benchmark images.

LPIPS, or learned perceptual image patch similarity, is a metric used to evaluate perceptual differences between images [25]. Unlike traditional metrics, such as mean squared error (MSE), which measure pixel-level differences or structural similarities, LPIPS employs deep learning to better align with human visual perception. In essence, LPIPS offers a more perceptually meaningful measure of image similarity, especially useful in tasks like image synthesis, where the objective is not just to reproduce pixel-accurate outputs but to generate outputs that are perceptually indistinguishable or pleasing to humans.

VGG distance refers to a perceptual loss metric based on the VGG network that was originally designed for image classification tasks [38]. Similar to LPIPS, it is used to measure the difference between two images in a feature space. The activations from one or more layers of the VGG network capture higher-level content and texture information about the images.

#### 4.3.2. Qualitative Results

We generate snow images in varying sizes by adjusting the previously discussed parameters and contrast our proposed model against the leading state-of-the-art methods.

In our experimental setting, we take the Cityscapes dataset and the EuroCity Persons dataset as the target set. These two datasets contain over 5000 thousand road scenarios under different urban and weather conditions. There are also variations of road users, such as pedestrians, cyclists, and moving vehicles.

**Figure 5:**
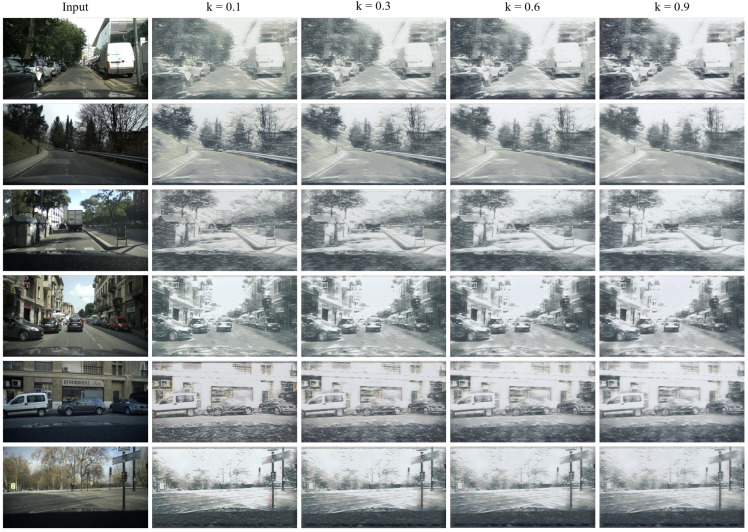
Synthesis of multidensity results on the EuroCity Persons dataset by adjusting the parameter *k*. From the left to the right column, objects such as vehicles, people, and trees are covered with snowflakes and haze that gradually increase in size.

Figure 5 displays images with varying amounts of snow. It is important to note that we assume that the k value of the input clear image is 0, while the value of the input snow image is 1. We then adjust the snow feature within the range of 0 to 1. The differences in snow density across images with distinct parameter values demonstrate our success in differentiating the generated snow through manipulation. As an illustration, as the value increases, objects at the far end of the image become less distinguishable. Due to the impact of the style feature coding, the style encoders can identify between large and small snowflakes.

**Figure 6:**
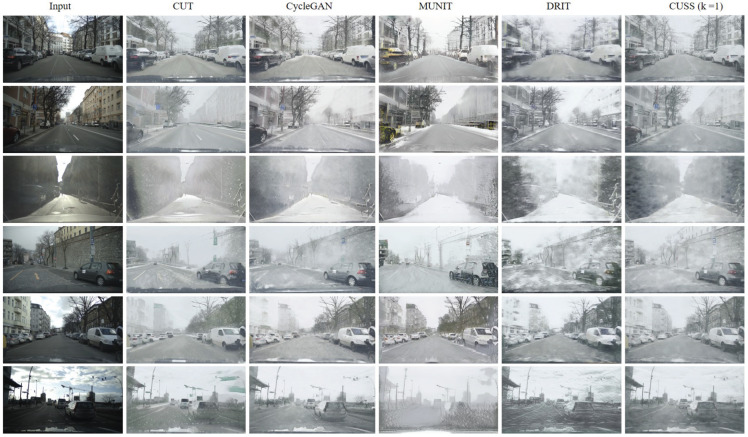
Comparisons between the synthesized snow images produced by our method and SOTA unsupervised image translation methods. In particular, CUT deviates from the utilization of cycle consistency and the associated loss, as observed in CycleGAN. Conversely, the remaining models, such as CUSS (controllable unsupervised snow synthesis), incorporate a form of partial style cycle consistency.

The results of the qualitative comparison are shown in Figure 6. This experiment compares the generated snow images with mainstream I2I translation methods (CycleGAN [14], CUT [40], MUNIT [40], and DRIT [26]). The first two models use single projections, while the last two can produce diverse outcomes. For a more accurate comparison, we consistently use ResNet [41] as the backbone for the generator in all methods. The training data use Cityscapes [34] and EuroCity Persons [35] as clear sources and the self-captured snow set as target sources. The methods used for comparison all require no paired data.

The qualitative comparison shows that models such as CUT [40], MUNIT [40], and DRIT [26] mainly exhibit three primary defects. First, after translating images to represent snowy scenes, the original colors are often distorted, diminishing the natural appearance of the scene. Second, these models sometimes introduce artifacts that were not present in the original image, leading to inconsistencies and jarring visual outcomes. Lastly, they inadequately handle the far end and sky regions, resulting in uneven or unrealistic snow representation in these areas. In contrast, the method proposed in this paper offers several advantages. Our approach naturally integrates snow, ensuring that its boundaries fade out seamlessly across the image, providing an authentic representation in both the foreground and background. By distinguishing between snow style and actual image content, our method is able to capture and reproduce the intrinsic properties of snow, resulting in a synthesis that feels genuine and consistent throughout the image. Moreover, while other models might render trees or other objects as if they were buried under un-natural snow formations, our technique retains the original structure and detail, providing a more balanced and realistic representation.

#### 4.3.3. Qualitative Results

As reported in other I2I works, the constraint of cycle consistency is strong so that the ability to generate diverse output is suppressed. However, the output image will retain a high similarity of the original image, which explains why CycleGAN [14] achieves the best SSIM value as shown in Table 1 and Table 2. Compared with other methods, CUSS combines the content discriminator and style code manipulation, and both turn out effective for high-quality synthesizing. Therefore, CUSS achieves better results on those metrics. CUT is the only method that does not employ any format of cycle generation pipeline, but instead uses contrastive learning. The data used in our experiment cannot satisfy the requirement of a large batch size, which cannot make full use of contrastive loss. The results of CUSS prove that our method is available even when the data are not sufficient.

To understand the individual contribution of different components of CUSS, we conduct an ablation study with respect to the loss functions. Since loss functions reflect the direction of model optimization, we not only validate the new module of the content discriminator and self-supervised style coding but also test the improvement from reconstructing the image, style code, and content code. In Table 3, we observe that each component is crucial to the CUSS model presented in the decrease of the metrics. We generate images with three sets of *k* (0.3, 0.6, 1). Smaller *k* values indicate the small size of the synthesized snow, i.e., closer to the input clear image. The results show that the model produces the best quality output at the smallest *k* values.

#### 4.3.4. Discussion

I2I translation methods such as CycleGAN, which uses the principle of cycle consistency, produce deterministic outputs. For a given clear image, it will produce the same translated snow image every time. To produce diverse outputs, researchers manipulate the latent space of extracted image features by dividing them into style codes and content codes. In our experiments, we found that latent space manipulation inevitably splits the translation network into two or more parts. This leads to performance degradation. In this work, the solution is to use a content discriminator to distinguish the content code from different domains. With the requirement of generating an indistinguishable content code, the encoder can achieve better disentangled representations.

When obtaining the disentangled style code, the operation on it will reflect on the output snow images [42]. Therefore, we interpolate the style codes of the input clear image and the snow image. Since the input snow image represents the maximum snow size, we can control the degree of snow effects. Note that we cannot obtain snow effects larger than the input image.

The controllable output is high quality and reasonable. The scenes are gradually covered with stronger snow effects. However, the generated snow is not invariant to objects. The snow covering the trees and the snow covering the building should be different; i.e., the snow effects should change appearance according to scenario changes. However, it looks similar in Figure 5. To improve CUSS, we need to consider the semantic information in the latent space.

Our method basically belongs to domain translation, which learns knowledge from the source domain and transfers it to the target domain. In the case of snow generation, the output will only contain a similar snow effect with input snowy images. To obtain more variety of snow like real snow scenes, we can add more snowy datasets that have different snow shapes and sizes. However, it will lead to mode collapse if there are too many modes in the GAN training process. In addition, the data should be collected in the same region to avoid large domain gaps, such as Asian driving scenes and European driving scenes.

## 5. Conclusions

The study presents an innovative approach to unsupervised snow synthesis, wherein a controllable method is introduced that incorporates latent space manipulation. To effectively separate the features of snow style and content, an additional content discriminator is incorporated along with a self-regression style coding module. To transition smoothly from clear to snow-affected images, a partial style cycle consistency loss is employed to refine the latent representation space. Furthermore, comparative analyses are conducted to comprehend the impact of each loss component or module within the model on the outcomes. When subjected to quantitative and qualitative evaluation, against various techniques using the Cityscapes and EuroCity Persons datasets, our approach consistently produces diverse and high-quality traffic scenes under snowy conditions. Moving forward, our future research endeavors can be classified into two distinct paths:

- Expanding the proposed technique to tackle generation tasks in more demanding driving conditions, such as heavy rain, dense fog, nighttime, and strong light;
- Delving deeper into the relationship between generative methods and latent space manipulation for I2I translation tasks by integrating existing insights from self-supervised and contrast learning methodologies.

## Figures and Tables

**Table 1:** A comparison on the Cityscapes dataset is made between SOTA image translation techniques through numerical evaluation. We generate images with k=1. The metrics used for evaluation are $d_{VGG}$ and $d_{LPIPS}$, which respectively represent the VGG and LPIPS distances. It should be noted that down arrows mean lower values for $d_{VGG}$ and FID indicate more favorable experimental outcomes. Conversely, up arrows mean that higher values for the other metrics suggest superior results. Optimal values are denoted in bold.

Methods	SSIM↑	PSNR↑	$d_{\mathrm{VGG}}↓$	FID↓	$d_{\mathrm{LPIPS}}↑$
CUT	0.392	15.853	6.063	26.156	0.047
CycleGAN	0.471	16.072	5.892	26.342	0.048
MUNIT	0.452	16.118	5.923	25.447	0.049
DRIT	0.446	16.432	5.426	25.874	0.049
CUSS	0.465	16.912	5.122	25.103	0.047

**Table 2:** A comparison on the EuroCity Persons dataset is made between SOTA image translation techniques through numerical evaluation. We generate images with k=1. The same image quality metrics are used. Down arrows mean lower metric values are better and up arrows mean higher values are better. The best results are shown in bold.

Methods	SSIM↑	PSNR↑	$d_{\mathrm{VGG}}↓$	FID↓	$d_{\mathrm{LPIPS}}↑$
CUT	0.396	15.912	6.123	26.245	0.048
CycleGAN	**0.501**	16.233	5.927	26.581	0.049
MUNIT	0.479	16.483	6.012	25.847	0.048
DRIT	0.469	16.741	5.756	26.007	0.048
CUSS	0.494	**16.983**	**5.386**	**25.402**	**0.051**

**Table 3:** Results from quantitative model comparisons after eliminating various loss factors are presented. We generate images with three sets of *k* values. We examine the impact of the content discriminator, cross-cycle consistency loss, reconstruction losses, and style regression loss. Down arrows mean lower dVGG and FID values signify improved experimental performance, while up arrows mean that higher values for the remaining metrics indicate better results. Figures in bold highlight the best values.

Module	SSIM↑	PSNR↑	$d_{\mathrm{VGG}}↓$	FID↓	$d_{\mathrm{LPIPS}}↑$
w/o $L_{id}^{x}$	0.459	16.868	6.044	26.156	0.046
w/o $L_{\mathrm{recon}}^{c}$	0.461	16.831	6.052	26.133	0.045
w/o $L_{\mathrm{recon}\;}^{s}$	0.466	16.843	6.032	26.092	0.045
w/o $L_{cc}$	0.462	16.857	6.063	26.112	0.046
w/o $L_{\mathrm{cont}\;}$	0.468	16.801	6.015	26.127	0.046
CUSS (k=1)	0.471	16.912	6.003	26.089	0.047
CUSS (k=0.6)	0.471	16.934	5.962	26.035	0.047
CUSS (k=0.3)	0.472	16.967	5.925	25.983	0.048

## Data Availability

Not applicable.
